# Lectures on the Operations of Surgery and on Diseases and Accidents Requiring Operations

**Published:** 1846-11

**Authors:** 


ART. II.—*Lectures on the Operations of Surgery and on Diseases and Accidents requiring operations.* By ROBERT LISTON, ESQ., F. R. S. *Senior Surgeon to the University College Hospital, and Professor of Clinical Surgery in the College. With uumerous additions, by* THOMAS D. MUTTER, M. D., *Prof. of Surgery in Jefferson Medical College, Philadelphia, etc. etc.* 1 vol. 8vo. Lea and Blanchard Publishers, 1846.

(Continued from page 270.)

*Nasal Polypy.* We have found so much difficulty at various times in detaching these tumors, notwithstanding we have varied our method and our instruments to almost every new suggestion, that we have looked anxiously to discover whether our author could give us in this matter any good advice.

"These tumors," says Mr. Liston, "are often found in great numbers. In this jar (presenting it) there are from a dozen to twenty. These tumors were all taken from the nostrils of one individual, at two or three sittings. These tumors generally adhere by narrow necks, have a smooth, moist surface, and I found, some years ago, that they were covered by ciliated epithelium. On placing a small shred, from the surface of a polypus under the microscope, soon after it is extracted, you observe the cilia in active motion, driving about the fluid which surrounds it, stirring the globules of blood about so as to make them present all their aspects. It is the only opportunity, I believe, you have of seeing the ciliated epithelium in action in the human subject. The substance of the tumour is soft, possibly a hypertrophy of the basement membrane, or of the submucus filamentous tissue. A few vessels will be observed ramyfying through it. Some of these polypous tumours, covered, as described, contain fluid. There is is a quantity of serosity in all tumours, even the most solid, which exudes when the surface is broken, or when they are punctured. But many such tumours are made up of cells containing serum. Sometimes the tumour consists of one large bag, or it is intersected, or several exist. The cysts are sometimes extracted unbroken, very often the fluid escapes during the operation, and the empty bag only remains to be taken away.

In cases of polypus, there is always a profuse discharge of mucus; the patient is constantly trying to use his handkerchief, but he cannot blow any air through the nostrils, or only imperfectly. The disease is attended with inconvenience, but it is not usually followed by any bad consequences. The disease, in other words, does not degenerate, and if you can take it away altogether, which is no easy matter, it is not, I believe, reproduced.

But there are other tumours met with here, which are of a very different nature, tumours which spring from the Schneiderian membrane, and fill up the nostrils very rapidly. They are attached by broad bases, and fill up the anterior and posterior cavities. Even if the disease is noticed from the earliest period of its commencement, you can do next to nothing to counteract it. You find, after a time, a large tumour projecting into the throat, and threatening to suffocate the patient. These tumours furnish a profusion of bloody sanious matter, and often a great quantity of blood. The disease is rapid in its progress, and may destroy the patient in a few months. Many of these tumors begin from the various sinuses connected with the nostril. They spring from the sphenoid and frontal bones, and sometimes grow from the surface of the nostrils themselves.

You find, now and then, a tumour projecting from the back of the nostrils, growing from the posterior nares, and hanging down the throat; some of the soft mucous polypi are found, though rarely, in that situation, and when the velum is raised up, they can be seen behind it. But, again, you find a fibrous tumour, very broadly attached, hanging down in this situation, and that tumour is not of a malignant kind. If you can succeed in removing the whole of it, it may not be reproduced.

Malignant tumours, I have said, are not to be interfered with; fortunately they are not of common occurrence, but the more you meddle with them the more rapidly the disease will be found to proceed. The soft mucous polypi are to be got rid of by the employment of the forceps—by evulsion. There is no use in trying to clip them off, for you will find it impossible to do more than to take away small portions of these swellings. There is no use in trying to tie them. I have seen that done over and over again, and when the polypus begins to shrink, the ligature comes away without bringing off the tumour. Besides, it would be endless work to apply ligatures to tumours when in such numbers, as I have shown you is almost uniformly the case."

This we took pleasure in marking as it afforded us great comfort, while recalling those awkward cases in which we have set down again and again to twist away polypi, which seemed to be reproduced as fast as they were detached, and we have at last given over the attempt to clear the nostril in despair. "You must twist them off," says Mr. Liston, "with proper forceps: small, delicate forceps —take hold with one pair to bring the polypus into sight, and *with another pair* seize it near its root."

"You must warn the patient that it will be impossible to cure him at one sitting, but you take away so many that he can breathe quite comfortably, and clear the emunctory, in other words, blow his nose. When there is a good deal of bleeding you cannot tell where other tumors are situated, or indeed whether there are any more. The patient will breathe freely for several days; perhaps there is some swelling, that goes down, and then the nostril appears clear, but within eight or ten days he comes back probably obstructed obstructed as much as ever. This does not arise from regrowth, but from the cavity of the nostril not being emptied. Some have been wedged in the anfractuosities of the cavity, these fall down and occupy the place of those taken away. You must deal with these in the same way as the former, and perhaps in three sittings you may remove the whole of them."

*Rhinoplastic operations.* Mr. Liston makes new noses sometimes, and in his own way — not very handsome always, he admits, nor very good noses for snuff taking, yet after all much better than putty noses or no noses at all. But *Dr. Mutter* works a good deal in this kind of plastic, ornamental surgery. It is a sort of hobby of his, which he rides very much of late;—yet it is true, as poor Yorick has said, that all men have their hobby-horses, "their running horses, their coins, and their cockle-shells, their drums and their trumpets, their fiddles, their pallets, their maggots and their buterflies"—we all ride some hobby (we have ourselves been accused of riding a pencil of nitrate of silver,) and we can not possibly see any objection to Dr. Mutter mounting the nose. Dr. Mutter has no superior in plastic surgery, and were he of Paris he would before this have received the decorations of the Legion of Honor, as a reward for his skill and his ingenious improvements in this department. But why, in the name of the Prophet, will he constantly occupy as much time in describing a simple operation for the repair of the bridge of the nose as was occupied by uncle Toby in building the draw-bridge of Namur? Here is a little hole in the side of the nose to be plugged up; the operation was *commenced* "*on the* 6*th of Oct. at* 12 *o'clock*" precisely, "in presence of Drs. Jackson, T. Harris, J. Randolph, J. R. Barton, P. G. Goddard, Langley"—Peter the porter and Betsy the chambermaid—for we beg to suggest, whether in the announcement of so important an event, every witness may not be considered material, and whether due weight has been given to the fact that both Peter and Betsy were directed to carry out the vessels containing the blood, and could swear better than all the doctors present as to the exact amount lost. The operation having been with due ceremony *commenced*, it only remained to be finished; but *when* it was finished, whether in one hour or six, does not appear. Nor have we any exact or approximate means of ascertaining, except from the fact that the description of the seating the patient, and seating the operator, and their several preliminary attitudes, together with the first, second, and third "cuts," with remarks, patients letter, etc. etc., occupies some six or seven octavo pages. Now Dr. Mutter does not claim that this operation is a "new invention," and that he has herein furnished a description for the patent office. The operation described by him *par glissement du lambeau* —is as old as the days of Celsus, and so vulgularly common is it that we have ourselves ventured to make the self same "cuts," and in the self same way, and have reported some of our doings at length in one of the early numbers of the first volume of this journal. We insist, therefore, that too much valuable paper is consumed by such lengthy reports. The accusation is a broad one, and we design it to apply not only to our friend Dr. Mutter, but to all who in publishing medical or surgical cases, attempt to build a world out of a mote, and describe the exsection of a wart as they would draw out a plan of circumvallation for a besieged city.

Who closes a *cleft palate*, must have skill and judgment beyond what belongs to one who merely "carves" well. Dr. Mutter has cured 19 out of 21 cases in which he has operated.

"It appears to me that the first thing to be done in our attempts to simplify the operation, is to show by positive results that it may be performed with more rapidity and a greater prospect of success by instruments such as I have described, which are always at hand, or even by others more simple, than with the assistance of all the special and complicated ones that have ever been invented."

"All the clamps to seize and hold the edges of the fissure, the crooked scissors to pare away the edges, the complicated "portes" for the needles, and "serre nœuds" for tightening the knots of the ligatures, corks to hold open the mouth, &c. &c., may, in most cases at least, be dispensed with, and not only this, but dispensed with to advantage."

Dr. Mutter requires merely "a knife, hook, long forceps, plain porte and needle, ligatures, scissors, sponges on long handles, cold water, wine and water, &c." The patient being prepared by frequent touching of the palate for some days previous, and by a mild cathartic, the operation is commenced by an assistant seizing the lower margin of the cleft, and rendering it tense, while the operator cuts with a short bladed knife from below, upwards to the apex of the fissure. The assistant still holding upon the long line of margin now detached, continues to pull moderately, while the operator carries the knife along the opposite margin to its lower edge. The second step is the introduction of the ligatures, which is accomplished in the usual way, with a very simple porte. The third step consists in tying the ligatures, which may be done better with the fingers than with a porte nœud, and cutting them off close to the knot.

*Age.* It is hardly safe to undertake this operation before the 16th or 18th year.

*Health.* He must be well and sound altogether, in his head and in his body; in his arteries and in his veins; in his fauces, in his pharynx; in his windpipe and in his gullet; in his ———

*Season.* "Mid-winter or late spring, probably answers best"—as also one should gather misletoe when the moon is six days old, and pluck up vervain at the rising of the dog-star! But we are of those infidels who believe that it matters little whether an operation is made when the moon is in the falx or the full—in the summer or the winter—when swallows leave their holes, or frogs are about to spawn, provided always the patient has a house, and knows how to make himself comfortable in it.

*Preparation of the Patient.* "If he be *in good health*, the administration of a purge the day before the operation is to be performed, will be all that is required"—to make him sick! certainly more than is required to make him well, and the advice can be considered as only one of those ill-timed concessions to a mischievous *custom*, which are too often made. The very best preparation—*always* the best, for any operation is *health*, and when that exists, "throw physic to the dogs."

*Difficulties.* To keep the mouth open and the tongue quiet. For either of which the stern will of the patient is better than any "apparatus major." Hemorrhage is a bagattelle.

The patient must neither sneeze, nor cough, nor hawk, nor speak, nor whisper, nor swallow, and if the irritation be too great for quiet endurance, he must be narcotized by injections. If he is hungry he must meditate upon the cold custard and calves-foot jelly, which he shall see on the third or fourth day. If he is thirsty he must wet the roof of his mouth with a damp sponge, or pour a thimblefull of water upon his tongue. If violent inflammation sets in, we may loose our patient, as did M. Roux, in 1836. The lencet is, of course, the chief anchor.

*Results.* The patient does not generally immediately experience any relief in his speech or deglutition, but after a time he will in both. But that most annoying circumstance, the passage of the food into the nostrils, is at once remedied.

*Tumours of the face.* "Erectile" tumours.—"Excision has been attempted in tumours of this kind in children, and sometimes with lamentable results. I think I have mentioned one case to you formerly, in which a practitioner of surgery attempted the operation of cutting out a tumour of this nature in his own house, and the patient died before he could be removed,—died, in fact, on the table, or in the nurse's lap—a very awkward sort of occurrence. You can sometimes extirpate erectile tumours very safely and advantageously by a combination of incision and ligature. By cutting round the tumour, which you can sometimes do without danger, and then tying it, you ensure its rapid destruction, save the patient considerable pain, and abridge the period of cure very materially. You cut through all the skin that is involved, in the first instance, or you pass a ligature under the tumour, and then, before tightening it, with sharp scissors, or a small knife, you make an incision between each two parts of the ligature, and tie it into the fissure, and in this way strangulation is sure to follow instantly. If you do not tie the mass very tightly at once, it becomes inflamed, and it may or may not perish from inflammatory action. This is always a painful and tedious process; but if you strangulate it at once, its destruction is instantaneous. It is painful at the time, but the aggregate amount of pain is far less than where mortification of the part takes place in consequence of inflammatory action."

*Cancerous tumours of the jaw.* "Some young surgeons have tried to signalize themselves by taking away tumours of this kind. I have known a great fuss made more than once about extirpating the jaw where the bone has been involved secondarily in cancerous disease, where the operation was, to say the least, exceedingly unjustifiable. The operation must have been very painful and very bloody; it is a proceeding attended with great risk of life, without the most remote prospect of the patient being cured of the disease. They have, as might have been predicted, died more rapidly, more miserably, than if they had been let alone. Older surgeons, and of some experience, have, it is said, looked on during their butcheries, and countenanced them by their presence. They ought to have known better, and accordingly been ashamed of themselves."

*Ulcers of the cheek.* In which chapter we have some very extraordinary "melo-plastic" operations, with sundry "melo-dramatic" drawings and illustrations, quite equal to the best of Cruikshank: faces horribly disfigured, made comely by the magic scalpel of Dr. Mutter. Truly the Dr. has a plastic hand, and his patients are "like clay in the hands of the potter."

*Stammering.* "Some rascally operations have been practised on the tongue to cure stammering, such as cutting a wedge-like slice out of it, as you would out of a salted and boiled bullock's tongue at table, and bringing the edges together. These operations are not founded upon any sound physiological principles, and so far from being successful, some prove fatal, and even where patients have recovered, they have not spoken better."

"[Every surgeon, I believe, will unite with Mr. Liston in his opinion of the operations for stammering. They are truly "*rascally*," and with the exception of that practised upon the frenum liguæ, when this from its shortness prevents distinct utterance, all, notwithstanding their high reputation for a time, have been consigned without a single regret, to the oblivion to which they are so justly entitled. T. D. M.]"

If Dr. Mutter intends by his exception in favor of that "practised upon the frænum," to sanction the operation of cutting the genio-hyo-glossus and hyo-glossus, we shall join issue and demand a "scire facias." No just reason can be assigned for its performance, nor can any cases, we venture to affirm, be shown of its final success! and it ought at once to take its rank with all the others which the Dr. has so justly rejected. Whoever practices either the section of the muscles under the tongue, or the section of the whole tongue *a la* Dieffenbach, or the excision of the tonsiles or uvula *a la* Yeasley, or acupuncturation of the tongue *a la* Detmould, or division of the frænum epiglottis *a la* Brain, to cure stammering, will serve the world better as a "surgeon to old shoes," for "in respect to a fine workman, his is but, as you would say, a cobbler."

*Tumors—Affections of the ear—of the neck—œsophagotomy*, "not had recourse to once in a quarter of a century."—*Poisons in the stomach;* "you cannot push a full sized tube anywhere, except into the œsophagus." *Wry necks—Deformities from burns;* here again we are in Dr. Mutter's territory, and hideously distorted faces yawn at us and might well nigh fright us from our discretion — but that vis-a-vis to each ugly mask sits a handsome face, "without scar or wrinkle"—"spot or blemish," or any such thing—"the deformed transformed."

*Affections of the mamma.*—Extirpation of the breast for cancer. —"At one time this was the most common operation in surgery. I recollect the period when a week seldom passed over without the operation being performed two or three times in our hospitals; but now it is seldom had recourse to, and properly too, except in cases of non-malignant disease."

*Affections of the clavicle—axilla—aneurism at the bend of the arm, etc.*—*Accidents from blood-letting.* "The operation of blood-letting is not to be undertaken rashly, and when you perform it, it must be done in a proper manner. The wound must be made in the right direction, and you must manage it well afterwards, so as to procure healing by the first intention. I do not often bleed patients, but one of the last I bled died, in some measure from the effects of this supposed simple and trifling operation."

*Amputation of superior extremities.* Here is one of Mr. Liston's hobbies. The flap operation is the *only* operation — and where he cannot well defend the superiority of this mode, he will make no operation at all — and it is thus, we have no doubt, he was driven from the lower end of the femur; declaring, in some of his books, that no amputation should be made below the middle of the thigh, while every body else would have us cut as low as possible. But it matters little, we conceive, which of the "thousand and one" incisions is adopted, provided we always remember M. Louis' rule, "skin enough to cover the muscle and muscle enough to cover the bone."

*Excision of joints—tapping—excision of ovarian tumours.* "Some people do not hestitate to make a hole in the abdomen, put in their fingers, and feel what is there, strangely enough exemplifying what Hudibras says—

'As if a man should be dissected
To see what part is disaffected.'"

*Hernia—contractions of the leg and thigh—anchylosis—club-foot!* A volume in itself, and nearly all from the pen of Dr. Mutter. Dr. Mutter was one of the American pioneers in tenotomy, and long since published a book on club-foot, which was well received. These pages contain all that the author has learned since that time, and as he is not one of those who hold "vetustas pro lege" we are not surprised to find that he has made considerable improvement in the application of splints, machinery, &c.

"So much, then," says Mr. Liston, "for *tenotomy*, as it is called. This is a part of surgery which every one of you ought to study. There is no difficulty in it whatever, and great advantage will often be derived from the proceeding. Every well educated surgeon ought to be prepared for it. It is an operation unattended with effusion of blood, and if *carefully* performed, no bad consequences can possibly arise from it. I have heard of serious results from the division of tendons; inflammation and suppuration of joints have supervened, and it has been a question in some cases, I am told, whether the limb ought to be taken off to save the patient. I have heard, besides, of the tibial arteries being cut, and of nerves being interfered with; but with proper management and care there need be no apprehension of such accidents occurring."

"*Calculous complaints*" close the volume. Some have thought that "stone in the bladder could be dissolved by medicines taken by the mouth." Thus Mrs. Stevens instructed the good people of London, and the British Parliament witn commendable gallantry, purchased the secret. But neither the cures, nor her ability to "keep the secret" until it was paid for, are more marvellous than the remedy itself—"soap and egg-shells!" The cure for hydrophobia, sold for $2,000 by John M. Crous, to the most wise and charitable Legislature of New-York, in 1802, was not a more divine gift,—"the jaw-bone of a dog pulverized, the false tongue of a newly foaled colt!" etc. And now that both the Waters of Vichy, and the Egg-shells of Mrs. Stevens have ceased their marvels, why do not our representatives in "Assembly and Senate convened" enlighten and "dissolve" the suffering thousands of this country, by the purchase of "Vaughn's Vegetable Lithontriptic," than which no solvent since the days of Alchemy has possessed one half the fame.

"Depend upon it," says Mr. Liston, "there is no way of getting quit of a stone after it has attained too large a size to come away by the urethra, without the application of instruments." "Tot homines — quot sententiae." F. H. H.

